# Plasma and Aorta Biochemistry and MMPs Activities in Female Rabbit Fed Methionine Enriched Diet and Their Offspring

**DOI:** 10.1155/2017/2785142

**Published:** 2017-01-04

**Authors:** Khira Othmani Mecif, Souhila Aouichat Bouguerra, Yasmina Benazzoug

**Affiliations:** ^1^Laboratory of Cellular and Molecular Biology, Biochemistry of Extracellular Matrix Remodeling, Faculty of Biological Sciences, University of Technological Sciences Houari Boumediene, BP 32, El Alia, 16011 Algiers, Algeria; ^2^Laboratory of Physiology of Organisms, Team of Cellular and Molecular Physiopathology, Faculty of Biological Sciences, University of Technological Sciences Houari Boumediene, BP 32, EL Alia, 16011 Algiers, Algeria

## Abstract

This study investigated whether a high Met diet influences biochemical parameters, MMPs activities in plasma, and biochemical and histological remodeling in aorta, in both pregnant female rabbits and their offspring. Four female rabbit groups are constituted (each *n* = 8), nonpregnant control (NPC), pregnant control (PC) that received normal commercial chow, nonpregnant Met (NPMet), and pregnant Met (PMet) that received the same diet supplemented with 0,35% L-methionine (w/w) for 3 months (500 mg/d). All pregnant females realize 3 successive pregnancies. Plasma results showed that Met excess increased Hcy, raised CRP in NPMet and decreased it in PMet, enhanced significantly proMMP-2 and proMMP-9 activities in NPMet, and reduced them in PMet. Aorta showed a rise in collagen level, essentially in PMet, a reduction of elastin content in both PMet and NPMet, and a significant decrease in lipid content in PMet, with histological changes that are more pronounced in NPMet than PMet. Met excess enhanced proMMP-9 activities in NPMet while it decreased them in PMet. PMet newborn presented increase in uremia and CRP and significant rise of active MMP-2 and MMP-9 forms. In aorta, media and adventitia thickness increased, total lipids content decreased, proMMP-9 activity decreased, and proMMP-2 activity increased.

## 1. Introduction

Met enriched diet causes hyperhomocysteinemia (Hhcy), which is associated with the metabolic syndrome, oxidative and nitrosative endoplasmic reticulum stress [1], inflammation, unfolded protein response, cell death, and increased cardiovascular risk [2].

Methionine, a sulfur amino acid with essential roles in intermediary metabolism, is a universal methyl donor for more than hundred reactions [3]; this amino acid is the only known source for homocysteine in mammals. Excessive methionine (Met) uptake might lead to Hhcy [4, 5]. Diet with Met overload might lead to the various pathophysiological consequences associated with Hhcy, such as hepatic lesions [6]. Some studies have reported that Hhcy caused structural and functional disorders in the aorta of experimental animals [7, 8]. Hhcy increases the risk of myocardial infarction, cardiovascular morbidity and mortality, and cerebrovascular disease [9–11]. Hhcy causes a stimulation of conversion of phosphatidylethanolamines into phosphatidylcholine and lipid deposits in the aorta [12, 13]. Hhcy induces HDL changes through Hcy-thiolactone, with loss of their anti-inflammatory and cytoprotective properties [14], and promotes LDL oxidation and internalization by macrophages, which is the initial step of atherosclerosis [15]. Considered by some authors as an important independent risk factor of atherosclerosis, Hcy can influence the atherosclerosis process in various aspects, through transmethylation, endothelial injury, inflammation response, and oxidative stress [16]. The Hcy autoxidation causes endothelial dysfunction, considered by certain authors, as the main initiator of atherogenesis, via H_2_O_2_ [17] with reduction of endothelial relaxation by fat accumulation inactivating cofactors of NOS, at the aortic wall. NO inhibits so platelets and leukocytes activation and maintains the SMCs to the nonproliferative state [18]. Akasaka et al. [19] noted the influence of Hhcy on chemotaxis of SMCs via the p30 protein as their migration from the media, playing a critical role in the expansion of the intima; the contractile SMCs pass from a quiescent to a proliferative state. Endothelial antithrombotic function of Hhcy is impaired by increasing activity of factors V and XII and reducing the activation of C protein [10, 20]. Hhcy induces dysfunction of global DNA methylation in blood vessels, via LOX-1 gene hypomethylation [21]. Excess of Hcy increases the synthesis and accumulation of collagen [22] and raises IL-6 production and cell adhesion of monocytes [23]. Under effect of Hhcy, ECM remodeling is accelerated by synthetic MMPs production with arterial elastic fibers degradation by elastase activation (MMP-1, MMP-3, and MMP-9) [24]. Proinflammatory cytokines, TNF-*α*, IL-6, and IL-1*β* potentially stimulate the expression of MMPs in macrophages and vascular SMCs [25]. Met enriched diet increases, in aorta, the mRNA and expression of the molecules VCAM-1 [26]. The MCP-1 protein is widely expressed in the endothelium with macrophage infiltration [27].

Endothelial cells contain the two types of ER *α* and ER *β* receptor; estrogen that stimulates the release of NO can modulate endothelial function by receptor activation, gene transcription, and dependent MAPK endothelial NOS (eNOS) activation [28]. Chikani et al. [29] showed that female HDLs contained significant amounts of estradiol, stimulating eNOS, but nongenomic effects of E2 on eNOS were observed. Dimitrova et al. [30] indicated the beneficial effects of E2 on lipid peroxidation, SMCs proliferation, and haemostasis. Spencer et al. [31] reported a reduction of superoxide anion rate, generated by NADPH oxidase, under E2 effect. The hematopoietic cells (macrophages, lymphocytes, etc.) express estrogen receptors indicating potential involvement in the regulation of the immune response [32]. A beneficial effect of E2 on Hhcy reduction and on neointima formation was noted [33]. The E2 effect on adhesion molecules is exercised through the NF- КB factor [34].

Extensive experimental studies indicate that a suboptimal environment during fetal and neonatal development in both humans and animals may program offspring susceptibility to later development of chronic diseases [35]. Some studies on animal models indicate that nutritional imbalance during pregnancy enhances susceptibility to atherogenesis [36].

Rabbit is a suitable model to analyse the impact of fetal development in offspring and adult health because of its lipid metabolism which is similar to that of humans and its sensitivity to develop atherosclerosis [37]. The main goal of the present study was so to investigate the effects of a high Met diet during pregnancy, on the aorta extracellular matrix of pregnant rabbits and their offspring.

If maternal alteration is responsible for increasing fetal lesion formation and susceptibility to atherosclerosis later in life, then pharmacological or nutritional interventions in mothers during pregnancy that prevent or minimize the onset of these pathogenic events in the fetus would be implemented to improve the health of offspring.

## 2. Materials and Methods

### 2.1. Animals and Diets

This study involved female domestic white rabbits (6 mo. old; body weight 2,7–3,1 kgs) obtained from Algiers agricultural cooperative. The animals were allowed free access to diet and water and kept in wire-bottomed stainless steel cages. Our study is made on nonpregnant (control) and on pregnant rabbits that realized 3 successive pregnancies during this experimentation period. They were housed with normal temperature and light cycles. Experimental period extended from February to May (22–28°C). Female rabbits were divided into the four groups: (a) the control group* (NPC)* (*n* = 8) was fed a normal commercial chow containing alfalfa (41,80%), oat bran (28%), barley (23%), corn (2,7%), soja (3,5%) equivalent to 30,46% protein, 8,26% lipids, 44,05% total carbohydrate, 11% moisture, 5% crude ash, 1% vitamin mixture, and 0,1% methionine (Met) content; (b) the high-methionine* (NPMet)* group (*n* = 8) received a normal commercial chow supplemented with 0,35% L-methionine (w/w) for 3 months (500 mg/day); (c) the pregnant control* (PC)* group received normal commercial chow; and (d) the pregnant group fed with high Met diet* (PMet)* is submitted to the same conditions as* NPMet* group. All the females of the groups* PC* and* PMet* realize 3 successive pregnancies. D-L-Methionine and other chemicals were obtained from Sigma Chemical Co. (St Louis, MO, USA). The chows were stored at 4°C. Food intake was controlled periodically.

Newborns obtained from control and experimented females are weighted and their blood and aortic tissue collected for biochemical and histological studies.

All experiments were carried out in compliance with the guidelines of the Federation of European Laboratory Animal Science Associations (FELASA) following approval by the local Ethical Committee of the Sciences and Technology, Houari Boumediene University, Algeria.

### 2.2. Methods

#### 2.2.1. Blood and Tissue Samples

Every week, during a period of 3 months, all the rabbits are weighted and the blood was harvested (marginal vein) after overnight fasting, into dry, citrate, and heparin-containing tubes. Plasmas and serum obtained by centrifugation at 1500 ×g for 10 min were stored at −80°C. After 90 days of diet administration, the rabbits were killed by decapitation. Aorta (from aortic valve to renal artery) was immediately sampled; one fraction is snap-frozen in liquid nitrogen and stored at −80°C for biological evaluation, another was introduced in Folch solution for lipid determination, another was fixed in adequate aqueous solutions and embedded in paraffin for histological analysis, and one fraction was cut into small segments, performed under sterile conditions, and designed for zymographic analysis.

#### 2.2.2. Determinations in Serum

Serum Hcy levels were determined by FPIA method in IMX apparatus [38]. Serum glucose, total proteins, urea, triglycerides, and cholesterol were determined by method of Barham and Trinder [39], Henry et al. [40], Weichselbaum et al. [41], Trinder [42], and Roeschlau et al. [43], respectively. For this assay, serum lipids were extracted with chloroform/methanol (2 : 1, v/v) mixture [44]. The extracted lipids were separated by thin layer chromatography and estimated by the methods cited below.

The C reactive protein (CRP) was determined by turbidimetry (at 552 nm) according to Eda et al. [45] method. Blood lipoproteins were separated by gel electrophoresis measured by densitometry [46]. Measure of gelatinase activities, MMP-2 and MMP-9, was performed by zymography, a polyacrylamide gel electrophoresis (10%), with gelatin (1%, Sigma) in nonreduced conditions [47].

#### 2.2.3. Determinations in Aorta

*(1) Lipid Content.* Lipid content of aorta was estimated by method of Folch et al. [44], after separation by TLC.

*(2) Activity of Diffusible Gelatinases.* A fraction of aorta was taken rapidly after animal dissection, cut in small fragments, and incubated, in sterile conditions, in 96-well microplate (100 *μ*L/hole), with DMEM (penicillin, 1%) during 24 h, at 37°C. The middle containing enzyme was removed and frozen at −80°C for activity estimation and the fragments of aorta are weighted; enzyme activity was expressed by mg protein.

*(3) Collagens and Elastin Determinations.* Frozen aortas are homogenized in ice cold Tris/Triton buffer (50 mM pH 7,4) and submitted to cold centrifugation (12 000 ×g for 30 min); collagens level was estimated by the reaction of OH Pro with chloramine T [48]. Measure of elastin extracted of the pellet was determined by spectrophotometry after reaction with ninhydrin (at 550 nm).

*(4) Histopathological Study.* Aorta from female and newborn rabbit was fixed in Bouin's solution and in 10% buffered formaldehyde, dehydrated in increasing concentrations of ethanol, cleared in toluene, and finally embedded in paraffin wax. Sections 5 *μ*m thick were stained with Masson's trichrome, PAS, Picro-Indigo-Carmine, and Sudan Black B for histological studies. Mean cross-sectional areas of aorta were calculated by measuring at least 50 areas for each aortal sample.

*Statistical Analysis.* All data were expressed as mean ± standard error of the mean (SEM). Statistical analyses were performed using StatView 5.0 Software. Differences between groups were determined by performing one-way analysis of variance (ANOVA). Comparisons were made with Student's *t*-test. A *p* value of *p* < 0.05 was considered statistically significant.

## 3. Results and Interpretations

### 3.1. Body Weights Evolution

Under the Met effect, we notice a decrease of litter size (35%) and a reduction in the length of gestation (28 days instead of 30) according to Dasarathy et al. [49], who indicate that Hhcy, during pregnancy, is implicated in adverse outcomes such as spontaneous abortion and premature delivery. Females fed with Met excess showed increase of body weight (*p* < 0.05). The newborn rabbits submitted to Met have a smaller body weight than controls in the first pregnancy, but those of the second pregnancy have a body weight more important (Table 1).

### 3.2. Female Progenitor

#### 3.2.1. Plasma Biochemical Parameters

**Figure 1:**
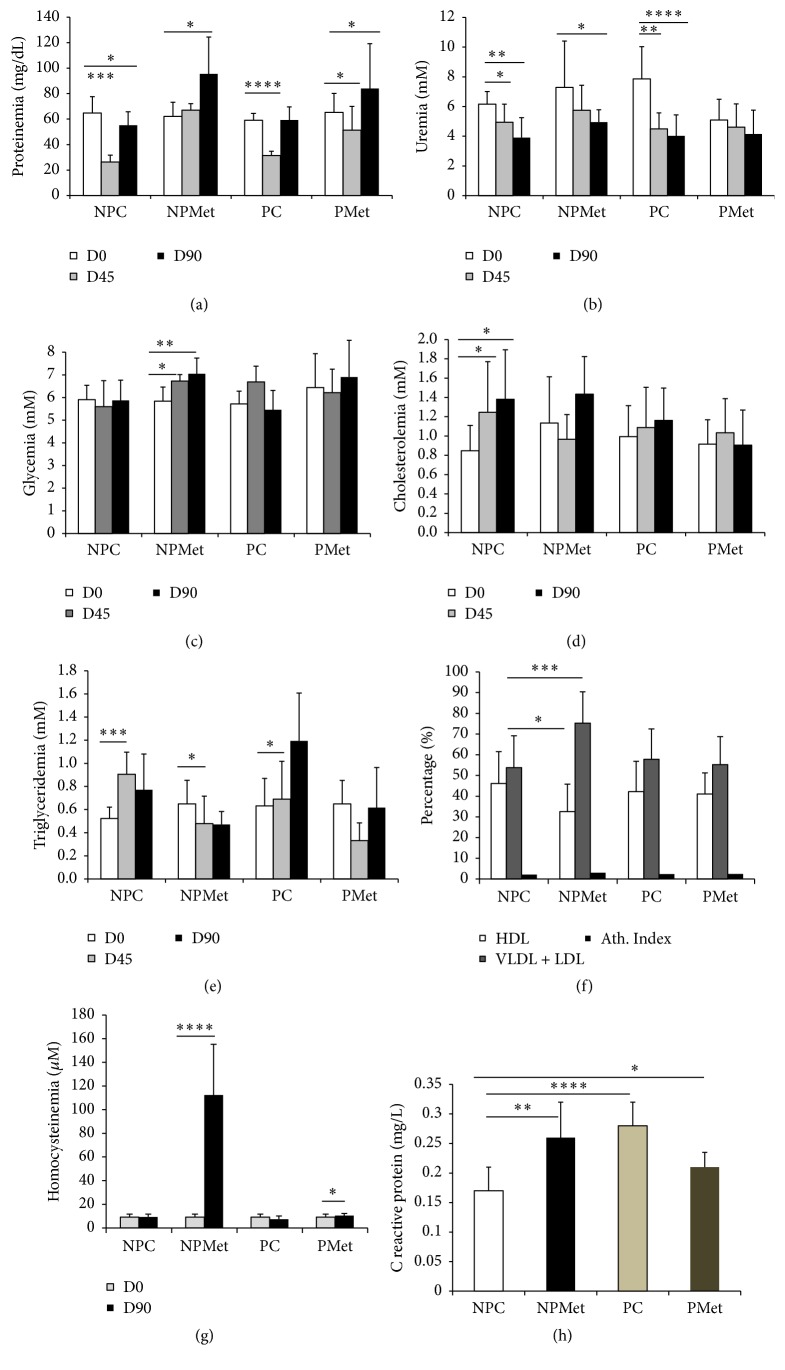
Plasma biochemical parameters of the 4 female rabbit groups. NP: nonpregnant control, NPMet: nonpregnant with Met, PC: pregnant control, and PMet: pregnant with Met. Comparison of NPMet versus NPC and PMet versus PC (^*∗*^*p* < 0.05, ^*∗∗*^*p* < 0.01, ^*∗∗∗*^*p* < 0.001, and ^*∗∗∗∗*^*p* < 0.0001).

Met diet administered to NP during 12 weeks increases plasma Hcy over 900% the initial value (*p* < 0.0001), while in PMet the elevation reaches 13,8%; pregnancy alone, without Met diet reduces Hcy in 30% (Figure 1(g)). Plasma CRP raises very significantly in PC (Figure 1(h)) and Met increases this parameter in NP rabbit (42,10%, *p* < 0.01) while decreasing this setting in P rabbit (16,66%, *p* < 0.05).

The fall in proteinemia, observed on the 45th day for PC group, is attenuated under the effect of Met with a recovery at the end of the study (Figure 1(a)). Uremia, which increases during a normal gestation, is paradoxically reduced with excess Met (Figure 1(b)). Glycemia and cholesterolemia do not seem affected by Met overload (Figures 1(c) and 1(d)). Triglycerides that increase in PC group in mid-experiment are reduced in PMet group (Figure 1(e)). The ratio of plasma lipoproteins *β*/*α* which is 2.17 in NPC increases significantly under the effect of Met (*p* < 0.01) (Figure 1(f)).

#### 3.2.2. Plasma MMPs Quantification

**Figure 2:**
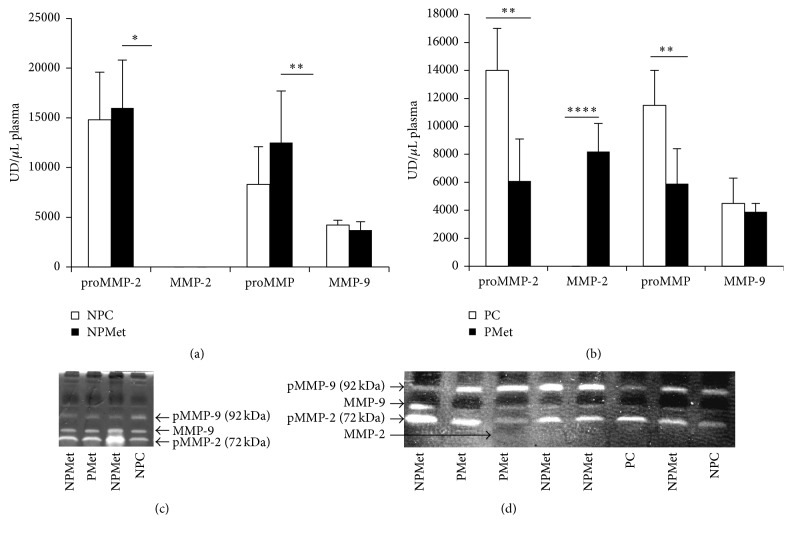
Plasma MMP activities of the 4 female rabbit groups. NP: nonpregnant control, NPMet: nonpregnant with Met, PC: pregnant control, and PMet: pregnant with Met. Comparison of NPMet versus NPC and PMet versus PC (^*∗*^*p* < 0.05, ^*∗∗*^*p* < 0.01, and ^*∗∗∗∗*^*p* < 0.0001).

Quantification of female plasma metalloproteinases reveals the presence of several forms of MMPs, such as proMMP -9, proMMP -2, active MMP-9, and MMP-2. The latter form is found only in the PMet group (Figures 2(b), 2(c), and 2(d)). Met enriched diet raises significantly proMMP-2 and proMMP-9 activities in NPMet and reduces them in PMet (*p* < 0.01) (Figures 2(a) and 2(b)).

#### 3.2.3. Aorta Biochemistry

**Figure 3:**
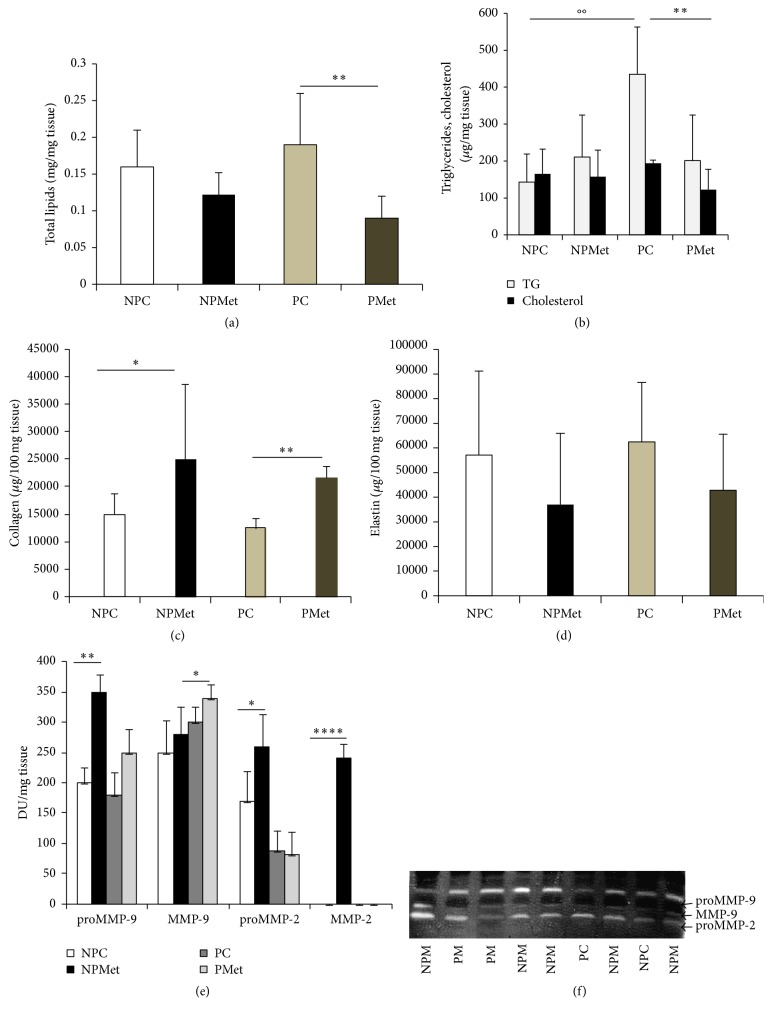
Lipid, collagen, and elastin levels and MMP activities of aorta from different female rabbits groups. NP: nonpregnant control, NPMet: nonpregnant with Met, PC: pregnant control, and PMet: pregnant with Met. Comparison of NPMet versus NPC and PMet versus PC (^*∗*^*p* < 0.05, ^*∗∗*^*p* < 0.01, and ^*∗∗∗∗*^*p* < 0.0001). The symbol “∘∘” means that the comparison is made between pregnant control TG and nonpregnant control TG.

Quantification of aortic collagen showed an increase in the rate of this component under the Met effect, which is significant in NPMet versus NPC (*p* < 0.05) and in PMet versus PC (*p* < 0.01) (Figure 3(c)). Met reduces elastin content in the aorta in pregnant and nonpregnant rabbits, but the individual variations make this analysis not significant (Figure 3(d)).

In thoracic aorta, total fat does not seem to be changed during pregnancy (Figure 3(a)) with significant accumulation of triglycerides (*p* < 0.01) and reduction of cholesterol (Figure 3(b)). Met enriched diet reduces significantly lipid content in pregnant aorta (more than 30%) essentially in cholesterol (*p* < 0.05).

#### 3.2.4. Aorta MMPs

Met excess increases significantly the activity of proMMP-9 in NPMet (*p* < 0.01), while it causes a decrease of this proMMP-9 activity in PMet (*p* < 0.01) (Figures 3(e) and 3(f)). The activity of active MMP-9 does not seem to vary significantly regardless of diet and physiological state. The proMMP-2 activity shows the same variations as the proMMP-9 in NP groups, with increase in NPMet (*p* < 0.05) but a nonsignificant decrease in the PMet. It appears that supplemented-Met diet causes a rise in proMMP-9 and active MMP-9 activities of rabbit aorta, but this diet raises the proMMP-2 activity in NP and has no effect on pregnant female aorta; the active MMP-2 form appears only in NPMet aorta (Figure 3(e)).

#### 3.2.5. Aorta Histology (Figure 4)

**Figure 4:**
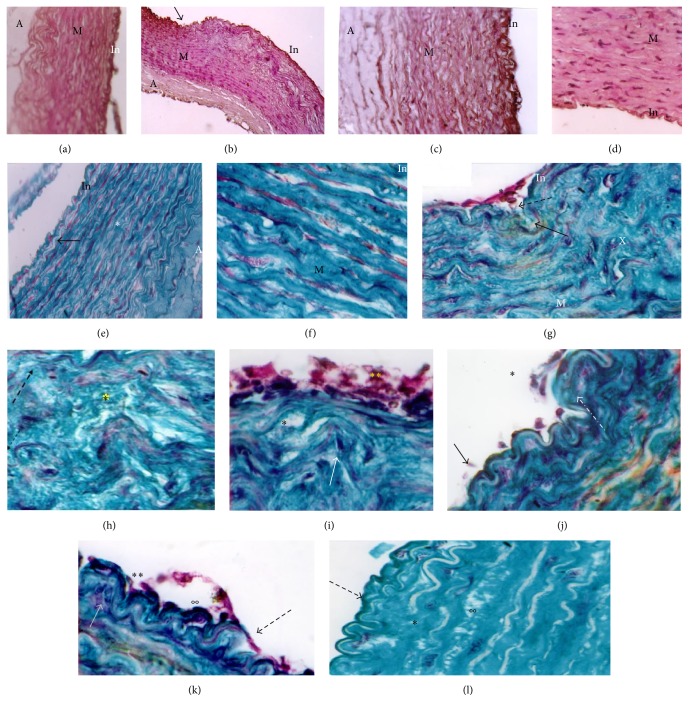
Aorta histology of the different groups of female rabbits. (a) NPC, (b) NPMet (×10 magnification), (c) PC, (d) PMet (×40) PAS staining; NPC ×40 (e); ×100 (f); NP Met ×100 (g, h, i, j, k, l) Masson's Trichrome staining. M: media; A: adventitia; In: intima. While control aorta shows regular elastic strips with regular intimal border (a, e, f), aorta of NPMet shows intimal thickening (b) and invagination areas, increasing contact surface. In (g, h), the SMCs appear in concentric organization and oriented towards the vascular lumen. We observe a complete disorganization of media elements, fragmented elastic strips, high ratio MEC/cells (*∗*), foam cells in media turbulence areas (*∗*), and occurrence of noncollagen material and thrombus (*∗∗*) (h, i). Intimal infiltration of SMCs, blood elements aggregation against endothelium (*∗∗*), internal elastic lamina fracture, hypertrophy of endothelial cells (dark kernels) (°°), duplication of internal elastic lamina, amorphous refractive material accumulation (°°), and endothelial cells hypertrophy (fingernail) characterize the aorta of NPMet.

**Figure 5:**
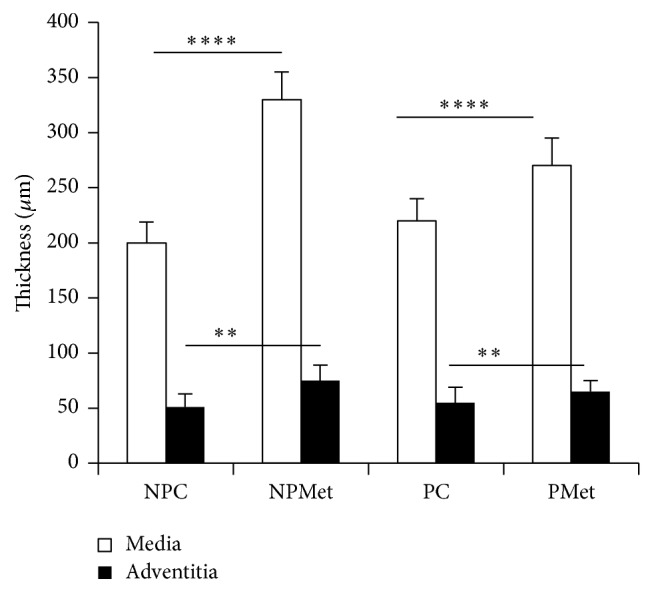
Aorta morphometry of the different groups of female rabbits. Comparison of NPMet versus NPC and PMet versus PC; ^*∗∗*^*p* < 0.01. ^*∗∗∗∗*^*p* < 0.0001.

**Figure 6:**
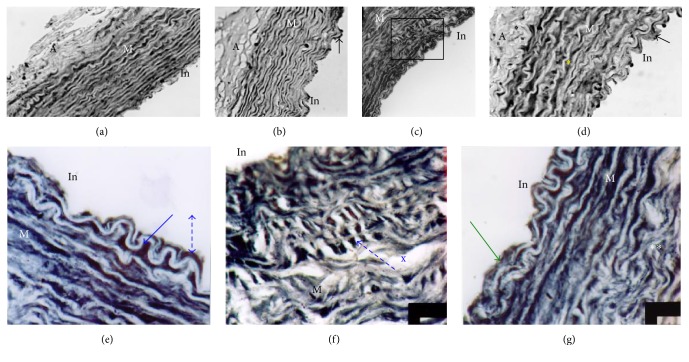
Aorta histochemistry of the different groups of female rabbits (Black Sudan B staining). (a) NPC, (b) PC, (c) NPMet, and (d) PMet. M: media, A: adventitia; In: intima; (a, b, c, d) are ×40 magnification; (e, f, g) are ×100 magnification of (c). (a) Control aorta. (b) Arrow shows blood cell agglutination. (c) The box indicates the area which is enlarged to (e, f, g). (d) Arrow shows endothelial elevation. (e) Lipid infiltration (arrow ) under intima and endothelial cell hypertrophy (dashed arrow) with important lipid load between blades. (f) Reorientation of media SMCs to the intimal direction (dashed arrow) and presence of amorphous material with diffuse appearance (X). (g) Intima thickness (arrow) with lipid load and collagen disorganization with elastic blades in tornado appearance (*∗∗*).

Aorta of NP rabbits, subjected to Met, undergoes severe histological changes, such as endothelium hypertrophy (cell nuclei as shaped nail), infiltration of SMCs and blood elements, presence of microthrombi fixed to the intima side, signs of impaired antithrombotic endothelium function (Figures 4(g) and 4(i)), and local rupture of the internal elastic lamina. Aorta media have profound remodeling, accumulation of collagens, disorganization and fragmentation of elastic blades (Figures 4(h), 4(k), and 4(l)), reduction in the cell/ECM ratio, change of SMCs orientation, which migrate to the lumen, and apparition of foam cells and amorphous chromophobic material. This de novo synthesis of collagens, combined with SMCs hypertrophy, leads to significant local thickening of the aortic wall. These changes are confirmed by morphometric analysis of NPMet aorta, showing a very significant rise of media thickness (*p* < 0.0001) and a significant elevation of adventitia size (*p* < 0.01) (Figure 5). The observed intima hyperplasia is local and unevenly distributed. In PMet aorta, we notice endothelial elevation with infiltration of lipid-rich cells in the intima (Figure 6(d)) and the presence of interlamellar lipid over the corresponding controls. Histochemical analysis, with Black Soudan B, indicates, in aorta of NPMet, hypertrophy of the endothelial cells, an important lipid load under intima, and in media, between the elastic strips, a presence of amorphous not sudanophilic material, which is combined with the fragmented elastic blades, gives a vortex structure aspect (Figures 4(h), 4(i), and 6(f)).

### 3.3. Newborn Rabbit

#### 3.3.1. Plasma Biochemistry

**Figure 7:**
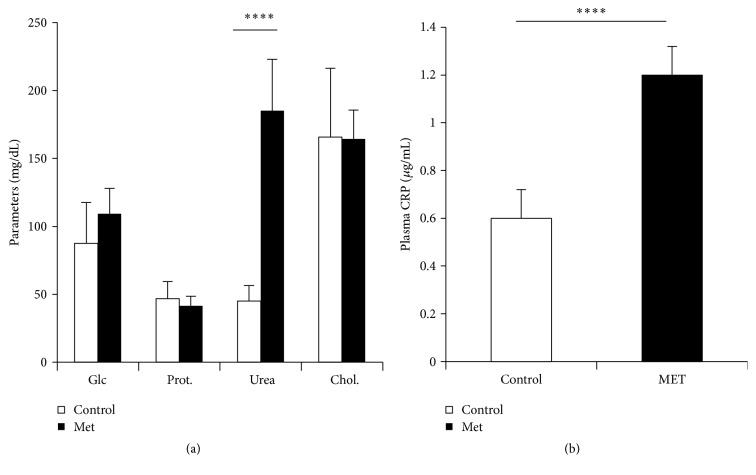
Plasma parameters of newborn rabbits. Glc: glucose, Prot: total proteins, and Chol: cholesterol. Control: offspring from control female rabbit (PC); Met: offspring from female rabbit submitted to Met (PMet); ^*∗∗∗∗*^*p* < 0.0001.

Biochemical parameters of newborn do not seem to be affected by Met enriched diet except for the uremia, which quadruples values (Figure 7(a)), and CRP that increases very significantly (*p* < 0.0001) (Figure 7(b)).

#### 3.3.2. Aorta Biochemistry and Histology

**Figure 8:**
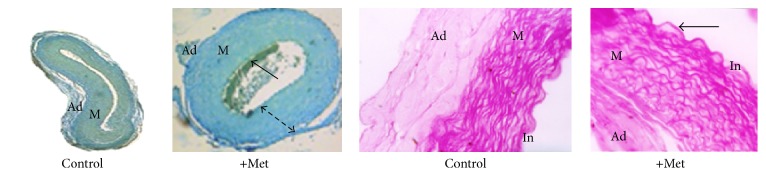
Histology of newborn rabbit aorta. Picro-Indigo-Carmine staining (×4 magnification) and PAS staining (×20 magnification). Under the effect of Met, the media thickness raises (dashed arrow); the* tunica intima* becomes apparent with endothelial elevation (→).

**Figure 9:**
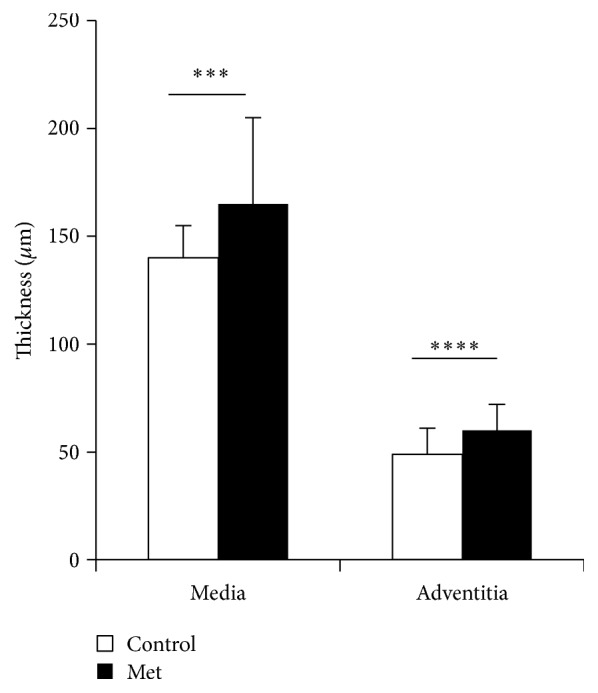
Aorta morphometry of newborn rabbit (^*∗∗∗*^*p* < 0.001; ^*∗∗∗∗*^*p* < 0.0001).

**Figure 10:**
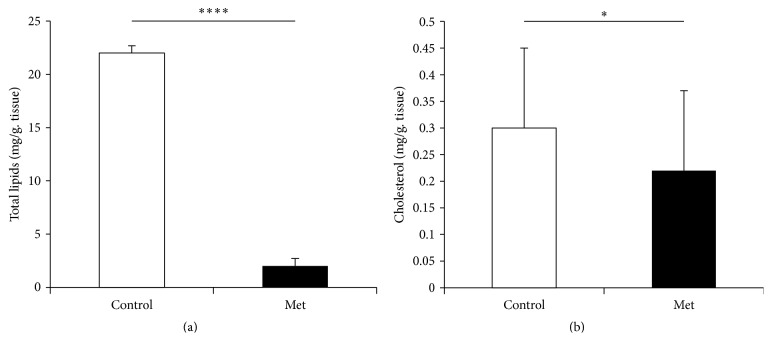
Aorta total lipids and cholesterol of newborn rabbit. Control: offspring from control female rabbit (PC); Met: offspring from female rabbit fed Met enriched diet (PMet) (^*∗*^*p* < 0.05, ^*∗∗∗∗*^*p* < 0.0001).

The aorta of newborn rabbit is modified under the effect of Met (Figure 8). Thus, we notice a marked aggregation of blood elements to the intima, endothelial elevation, and disorientation of the media SMCs. Morphometry measurements show a very significant increase in both the medial and the adventitia areas (Figure 9). Aorta total lipids content decreases very significantly (*p* < 0.0001) under the Met effect (Figure 10). This decrease does not seem to be attributed to the cholesterol.

#### 3.3.3. Plasma and Aorta MMPs Quantifications

**Figure 11:**
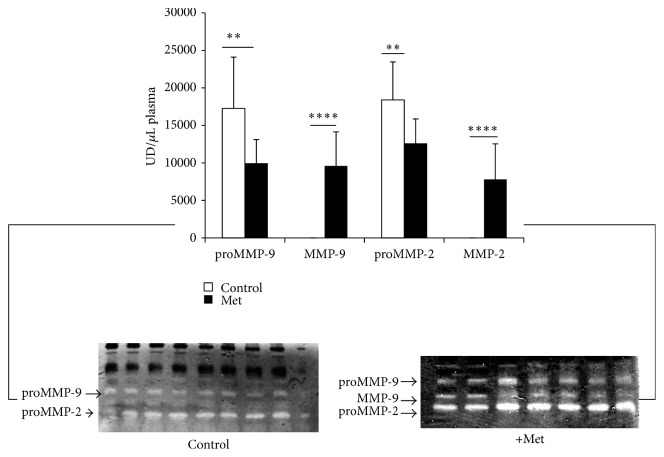
Plasma MMPs activities of newborn rabbit. Control: offspring from control female rabbit (PC); Met: offspring from female rabbit submitted to Met (PMet) (^*∗∗*^*p* < 0.01, ^*∗∗∗∗*^*p* < 0.0001).

**Figure 12:**
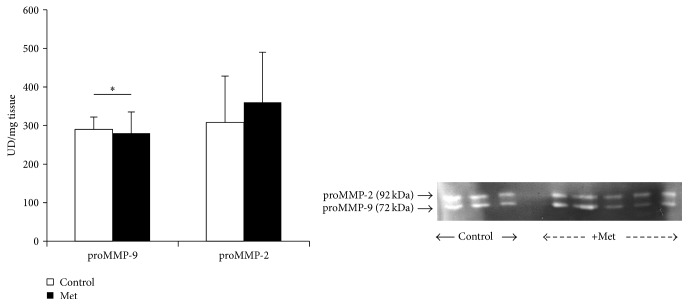
Aorta MMP activities of rabbit offspring. Control: offspring from control female rabbit (PC); Met: offspring from female rabbit fed Met enriched diet (PMet). (^*∗*^*p* < 0.05).

Newborn control plasma shows the 2 forms of inactive MMP-2 and MMP-9 but plasma Met newborn reveals significant rise of active MMP-2 and MMP-9 forms (*p* < 0.0001) with important reduction of the inactive forms (proMMP-2, proMMP-9) (*p* < 0.01) (Figure 11). It seems that only the proactive forms of MMP-2 and MMP-9 activities are apparent in both control and Met aorta, with significant reduction of proMMP-9 activity under Met effect (*p* < 0.05) (Figure 12).

## 4. Discussion

The current study demonstrates that excess Met diet affects the metabolism of Hcy; for Refsum and Ueland [50], it causes alteration in transsulfuration system and methylation leading to a state of hyperhomocysteinemia (Hhcy). The administration of Met enriched diet increases body weight of pregnant and nonpregnant rabbits with a significant increase for the latter. Thus, the weight of NP is very significantly higher (*p* < 0.0001) after a month of diet, although some authors [51, 52] did not observe variation of this parameter in pigs and rats, respectively, while other authors found a reduction in the body weight, such as Giroux et al. [13], on rabbits submitted to a mixture of Met-Lys, and Zerrouk [53], on sand rat (70 mg/d. for 6 months).

Administration of this diet, during 3 months, allows the development of important Hhcy in NPMet group (*p* < 0.0001); this increase is much greater than that obtained by Rolland et al. [51], on the pig fed with excess Met (3,45 g/day for 4 mo.) The rate of Hcy in PMet is slightly modified compared to controls (*p* < 0.05). The administration of Met causes a significant increase in CRP levels among NP (*p* < 0.01); this protein produced by the liver, adipose tissue, and monocytes/macrophages in injured areas would be responsible for oxidized LDL opsonization [54]. On the other hand, Met seems to reduce CRP in PMet. The inflammatory role of Hhcy is mentioned by many authors, such as Fang et al. [55], demonstrating that Hhcy promoted circulating inflammatory monocyte. So, Hhcy induces inflammatory MC differentiation leading to proinflammatory cytokine production (IL-6 and TNF) and systemic inflammation and raises chemokine (MCP-1) level [56]. Yang et al. [15] noticed high level of TNF-alpha and ICAM-1 expression in rats exposed to methionine-rich diet.

After 3 months of experimentation, excess Met appears to raise glycemia in both pregnant and nonpregnant rabbits (*p* < 0.01) (Figure 1(c)); this is not according to El-Wahab et al. [57], indicating that high-methionine diet can stimulate insulin secretion from pancreas. On the other hand, the glycemia increase in pregnant rabbits can be the consequent of gluconeogenesis, as this pathway can occur, during normal pregnancy, from ketogenic amino acids on liver and other peripheral tissues [58].

Excess Met in the NPMet causes variations in cholesterol which slightly exceeds that observed in NP control. For Sugiyama and Muramatsu [59], this effect is the consequence of sulfide groups since cystine (S–S) used in other experiments did not produce this action; Met turned into Hcy appears with 25% in free form, which is unstable and oxidized [60]. For Montoudis et al. [61], modification of cholesterol metabolism during rabbit gestation resulted in hepatic ACAT and HMG-CoA-reductase modulation, in both dams and offspring. Marseille-Tremblay et al. [62] showed the key role of placenta in cholesterol synthesis during pregnancy via SREBP-1/2 action.

According to Voutilainen et al. [63], plasma Hcy is quickly self-oxidized into homocystine, Hcy-thiolactone, and Hcy-disulfite; these authors note also that the Hhcy is associated with lipid peroxidation increase in humans. Other authors, such as Koyama [12] and Fujimoto et al. [64], suggest also a growth of cholesterol, TG, Hcy, Cys, and lipid peroxide in rabbits fed with Met enriched diet (3%, for 22 weeks and 4 months, resp.). Paradoxically, excess of Met reduces TG, as well as NPMet compared with PMet; these lipids would be taken up by the vascular wall as mentioned by Yang et al. [65], noting that Hcy leads to cholesterol ester and TG accumulation in the vascular wall of monkey.

Similarly, we notice, in NPMet, a lipoprotein (VLDL + LDL/HDL) ratio of 3.07 (limit 3) indicating that the NPMet rabbits are in atherosclerotic state; for PMet rabbits the ratio is 2.44 which underlines the protective effect of sex steroid hormones, mainly estradiol, on the vascular wall. This hormone increases in fact the plasma membrane fluidity, promoting exchanges between endothelial cells and lipoproteins. The HDL has antiatherogenic effects (cholesterol reverse transport), protects against the LDL oxidation (HDL 3), and presents anti-inflammatory, antiapoptotic, and antithrombotic actions [66].

The Met enriched diet administered to rabbits leads to a significant elevation of proteinemia in the NPMet and a moderate rise in PMet, suggesting that the Met excess is mainly drawn to the embryo-fetal development. The measured uremia shows an inverse evolution to that of total protein with significance for the NPMet.

Met administered in the diet appears to stimulate protein synthesis demonstrating the leading role played by the Met, donor of methyl groups in various synthetic ways [5], especially since Pappa et al. [58] noted a proteolysis stimulation during a normal gestation. For Dasarathy et al. [49], the early gestation period requires more Met for transsulfuration, whereas high transmethylation late in gestation period needs more methyl donors (betaine, folate).

Excess Met ingested is not metabolized in urea [67]; in human and mice the resulting Hhcy is transformed into thiolactone, an atherogenic metabolite, which is eliminated in the urine.

In plasma, the proMMP-2 and proMMP-9 are highly expressed in NPMet compared to PMet, and the active form MMP-2 is the predominant circulating form in PMet. This result suggests that excess of Met, and consequently the Hhcy, stimulates the expression of diffusible metalloproteinases produced by the cells blood and by the fibroblasts of different tissues including the aorta, in accordance with the rearrangements that we have observed.

It appears clearly that the diet supplemented with Met (500 mg/kg/d) causes disruption of the plasma biochemical parameters, contrary to the results obtained by Rolland et al. [51] and Augier et al. [68]. Rai et al. [1] demonstrated that Hcy caused ER stress and thus UPR (unfolded protein response) because it raised the GRP 78 (glucose-regulated protein), a molecular chaperone involved in the UPR.

For Yang et al. [69], Hcy might promote LDL oxidization and induce blood vessel global hypomethylation mediated by LOX-1.

Met excess reduces aorta total lipids especially in pregnant cases. While a significant reduction in TG and cholesterol in the aorta of PMet occurs, we note TG accumulation in the NPMet aorta, according to histochemical results (Sudan Blacks staining), which shows high chromophilic tissues (especially for NPMet). Thus, excess Hcy inhibits methylation of lipids and proteins of LDL, increasing their endocytosis [65]; secondly, the reactive oxygen species (ROS) released during the Hcy oxidation modify LDL; these oxidized or fixed to homocysteine bind to scavengers A and B. Their binding with SR A stimulates transcription and release of cytokine, engaging the inflammatory process. Oxidative stress generated by the Hhcy and LDL ox (ROS and carbonyl adducts) reduced the availability of NO and alter structures and functions of caveolae, which are transcellular transport system of various molecules, including amino acids (Met), in endothelial cells [70].

Hhcy causes methylation of L Arg to dimethyl Arg, an inhibitor of NO synthase (NOS). According to Cayatte et al. [71], lipid accumulation constitutes an inhibitor of NOS in the aortic wall. Miller et al. [72] mentioned reduction of NO rate in both endothelial and platelet levels. The endothelial dysfunction appears to be due to nitrosative and endoplasmic reticulum stress rather than oxidative stress or lack of eNOS [1]. Kruzliak et al. [73] showed that oxidized LDL induces ER stress via a LOX-1 pathway, with other stress pathways leading to endothelial dysfunction.

The intimal thickening, characterized by hypertrophy of the endothelial cells (lunar shaped cores) and an accumulation of subendothelial material, is also observed by Ichikawa et al. [74] in rabbits and Yang et al. [65] in mice subjected to excessive Met. According to Chen et al. [75] the intimal hyperplasia is stimulated via NMDAR receptor, which exists in peripheral vessels.

The increase of vascular adhesion, observed by us in rabbits fed with Met excess, is consequent to the expression and production of adhesion molecules by endothelial cells, such as VCAM-1 [26], E-selectin, and ICAM-1, the latter indicating the advanced stage of atherosclerosis [52].

We also noted hypertrophy of SMCs, their subendothelial infiltration, and their reorientation in the media; this hypertrophy was already noticed by Fanapour et al. [76] in vitro and Akasaka et al. [19]. These authors state that the Hhcy influences the chemotaxis of SMCs* via* the p30 protein; their migration contributes to enlarging the intima. The SMCs change from quiescent contractile state to synthetic proliferative state, producing the MMPs and accelerating matrix remodeling.

Our results showed an increase in collagen and a diminution in elastin as demonstrated by Steed et al. [77] indicating that Hhcy induces remodeling of the arterial wall ECM by induction of MMPs, consequences of several mechanisms in addition to oxidative activation. Zulli and Hare [78] mentioned the stimulatory effects of Hcy on collagen. The observed collagen deposition may contribute to the observed medial thickening such as that obtained by Steed et al. [77].

Reduction of elastin content measured at NPMet aorta reflects the fenestration we observed by histological analysis in aorta elastic strips of this rabbit group. The reduction of elastin level was also observed in chick aorta subjected to diet enriched with 2% of Met [79]. It seems that PGs are responsible for these anomalies [80].

The analysis of aortic MMP-2 and MMP-9 activities consolidates the biochemical and histological results. Thus, the proMMP-2 and MMP-9 that are absent in the two groups of control appear as a consequence of Met, with accentuation to NP indicating atherosclerosis status, their expression being stimulated in macrophages and vascular SMCs. The presence of aortic active MMP-2 form, which is observed only in the NPMet, indicates that elastin itself, by structural change, activated proMMP-2 as MMP-2 (contact activation) leading to its own autolysis with significant reduction in its content as a consequence.

For Narayanan et al. [81], Hhcy increases the expression and activity of MMP-9 and causes matrix degradation and accumulation of collagen in the ECM. During normal physiological remodeling, MMPs and TIMPs are in tight coordination to maintain optimal vessel wall structure, but Hcy elevation inactivates TIMP and therefore enhances MMP activity [77]. Steed et al. mentioned that an increase in iNOS activity was a key contributor to Hhcy-mediated collagen/elastin switch and decline in aortic compliance. As a consequence, Hhcy limits the bioavailability of nitric oxide (NO) and alters the elastic properties of vascular walls by increasing matrix metalloproteinase (MMP) activity. These authors suggest that Hcy has no effect on the regulation of MMPs at the gene or protein level but exerts the MMP regulation by other mechanisms such as cytokine-mediated and inflammatory processes. Another study found that arterial remodeling, observed in an animal model of Hhcy-induced arterial hypertension, was in part due to increased MMP-2 and MMP-9 activation [34].

It is demonstrated that MMP-2 and MMP-9 facilitate cell movement by breaking down the basement membrane and degrading type IV collagen; many MMPs seem to control, with precision, processes of immunity, cell migration, and angiogenesis via a variety of extracellular effector proteins, including cytokines, chemokines, and some ECM components [82].

For Chaussalet et al. [83], the endothelial cells are involved in the proteolytic degradation of subendothelial matrix by increasing MMP-2, MMP-9, and human tissue kallikrein (HK1). MMP-9 induced by Met overload is the target of the IL-12, a cytokine produced by macrophages, an intermediary in the dialogue blood cells-immune cells, producing several cytokines (IFN *γ*) by T and NK cells [84]. Bischof et al. [85] indicated that MMP-9 secretion was under the control of ECM glycoproteins, hormones, cytokines, and growth factors.

The proMMP-9-NGAL complex we get on NPMet aorta reflects the significant vascular remodeling by these metalloproteinases modulated by neutrophil gelatinase; similar cases described by Hemdahl et al. [86] occur during hypoxic stress (10% O_2_). Pregnant rabbits subjected to the Met exhibit low Hcy and so stimulation of these MMPs is reduced, and more these enzymes exhibit a significant reduction in their activity.

In pregnant rabbits, the Met excess has little effect on the plasma biochemistry and on the components of the target tissues; this is the consequence of high circulating estradiol levels that reduce hyperhomocysteinemia as demonstrated by Lacut et al. [33], reducing the production of adhesion molecules* via* inhibition of NF-КB and inhibiting the formation of neointima [72].

In addition, these hormones exert their neutralizing function on reactive oxygen species through the phenol cycle, as scavengers of free radicals. These hormones exert also their effect by stimulating the synthesis of glutathione and antioxidant systems, SOD, catalase, thioredoxin reductase, GPX, and GST, the latter being in the respective control of estradiol and progesterone [87].

Met overload has also an impact on the rabbit offspring. Maternal nutritional status may result in alteration of the epigenetic state of fetal genome (alteration of the DNA methylation, histone modifications) leading to lethal changes resulting in litter size reduction as we have observed. We note that some females present a reduction of pregnancy duration, from 30 to 27 days and others present outcomes. This result is in agreement with that found by Eskes [88] who showed that outcome occurs by rupture of uterine spiral arteries, consequence of the combination Hhcy-thrombotic factors (C protein, S protein, antithrombin, and Leiden factor V).

The histomorphological changes observed in aorta of NP rabbit, fed with Met enriched diet, such as intima hyperplasia, media remodeling, collagen accumulation, elastic blades disorganization, and fragmentation, have been mentioned by other authors [73, 78], but effect of Met, on pregnant rabbit and newborn aorta, has not been studied.

Met appears to influence body weight of newborn rabbits, with a significant reduction of this parameter in the first pregnancy and an increase in the second one. Alteration in the glucose availability could endanger fetal development and could explain the reduction of fetal birth weight [89]. On the other hand, the amino acid glycine is required for the catabolism of excess dietary methionine, so some long-term effects of excess methionine may be the result of glycine deficiency and may also cause trouble in fetal development [90].

The plasma parameters are unchanged except uremia which is significantly high (*p* < 0.0001). With glucose and amino acids being preferred metabolites in the fetus, it seems that excess Met is transferred to the fetal compartment. Thereafter, Met or Hcy are converted to Glc via gluconeogenesis with significant production of urea.

These newborn rabbits exhibit an inflammatory response, since their plasma CRP is dramatically elevated, indicating that Met excess is an aggression for these new tissues, such as aorta, which presents local thickening as observed in their progenitor. For Bellisario et al. [91], the environment experienced in utero does not only determine the growth trajectory of the fetus, but also contribute to disease susceptibility in later life.

Apparition of the active circulating forms MMP-2 and MMP-9 highlights the phenomenal remodeling that occurs under the influence of excess Met. Furthermore, in aorta, the presence of only the zymogen forms (proMMP-2 and proMMP-9) and the reduction of proMMP-9 rate mean that the anabolic process exceeds the catabolic phase.

Although lipemia is unchanged, we find a very significant reduction of total fat and cholesterol in aorta; Met seems to prevent absorption and integration of lipid in this tissue.

The histomorphological, histochemical, and enzymatic studies of rabbit aorta, submitted to Met confirmed the development of atherosclerosis process in rabbits NPMet. Therefore, in plasma of Met fed model the evolution of proMMP-2 and proMMP-9 is similar, that is, highly expressed in NPMet; they are underexpressed in PMet; this reduction can be explained in part by inhibition of NOS, as noted by some authors [72]. In fact, during the pregnancy, the estrogen level (ovary, placenta) increases NO production by different mechanisms. Our study shows the reducer effect of female hormones on Hcy.

## 5. Conclusion

This study shows that Met-supplemented diet causes large plasma variations (lipids, Hcy, and CRP) and ECM connective tissues remodeling in aorta (collagen, elastin, rise of proMMP-9, proMMP-2, and apparition of active MMP-2) of pregnant and nonpregnant rabbits, with a more pronounced effect in nonpregnant cases. This means that the NO bioavailability or content is little or not reduced by pregnancy. During pregnancy the significant rise of estrogen level increases the NO production by different ways, via the eNOS and iNOS of the vascular endothelium luteum [92] and those of placental and uterine blood vessels and via nNOS of the vessel adventitia [93].

Maternal nutritional status may result in an alteration of the epigenetic state of fetal genome (steady changes in gene expression) induced by alteration of the DNA methylation and histone modification [90]. The rabbits of PMet group present a small increase in Hcy (*p* < 0.05); their offspring are leaner than those of control dam but their agility is not altered, with normal behavior, indicating a normal maturation of their brain, since Baydas et al. [94] mentioned a cerebral immaturity in offspring of maternal Hhcy. Biochemistry of newborns is also affected by Met enriched diet; in both plasma and aorta tissue, they exhibit an inflammatory response, since the CRP is highly elevated, indicating that Met excess is aggression for these new tissues irrigated by an inadequate environment, with a very significant reduction of total lipids as it was observed in their progenitor, indicating also that Met seems to prevent the absorption and integration of lipids in aorta tissue.

## Figures and Tables

**Table 1:** Female and newborn body weight (gr).

	D_0_	D_90_	Significance
NPC (*n* = 8)	2902 ± 191	3008 ± 253	NS
NPMet (*n* = 8)	2461 ± 112	2851 ± 283	^*∗*^ *p* < 0.05
PC (*n* = 8)	2705 ± 154	3098 ± 176	^*∗∗*^ *p* < 0.01
PMet (*n* = 8)	2781 ± 214	3042 ± 132	^*∗*^ *p* < 0.05
PC newborn (*n* = 45)	50 ± 4		
PMet newborn (first pregnancy) (*n* = 22)	36 ± 7		^*∗*^ *p* < 0.05
PMet newborn (second pregnancy) (*n* = 20)	62 ± 15		^*∗*^ *p* < 0.05

NPC: nonpregnant control, NPMet: nonpregnant + Met, PC: pregnant control, and PMet: pregnant + Met.

Comparison of females body weight D_90_  versus D_0_ and PMet newborn body weight versusPC newborn body weight. ^*∗*^*p* < 0.05; ^*∗∗*^*p* < 0.01.

## Abbreviations

ER: Estrogen receptor
Hcy: Homocysteine
iNOS: Inducible nitric oxide synthase
Met: Methionine
MMP: Matrix metalloproteinase
NO: Nitrogen monoxide
OH Pro: Hydroxyl proline
ROS: Reactive oxygen species
SD: Standard deviation
SMC: Smooth muscular cell
TG: Triglycerides.
